# Preliminary findings on the effect of the pig appeasing pheromone in a slow releasing block on the welfare of pigs at weaning

**DOI:** 10.1186/s40813-016-0030-5

**Published:** 2016-06-01

**Authors:** Déborah Temple, Héloïse Barthélémy, Eva Mainau, Alessandro Cozzi, Marta Amat, Maria Eugênia Canozzi, Patrick Pageat, Xavier Manteca

**Affiliations:** 1 School of Veterinary Science, Universitat Autònoma de Barcelona, Bellaterra, 08193 Spain; 2 IRSEA (Research Institute in Semiochemistry and Applied Ethology), Quartier Salignan, 84400 Apt France

**Keywords:** Pheromone, Mixing, Pigs, Behaviour, Weaning

## Abstract

The pig appeasing pheromone (PAP) applied in spray has shown to be effective in reducing the frequency of aggression and the stress response of young and adult pigs under experimental conditions. This preliminary experiment investigates the effect of the PAP in a slow releasing block on the behaviour and skin lesions of weaners after mixing on a commercial farm. Two identical rooms containing six replicates per treatment of a commercial weaner building were used. There were two treatments (PAP block and Control) and each room contained one treatment. In the PAP treatment, the day before weaning and after washing and disinfection, two PAP blocks (1 block / 20 m^2^) were placed hanging on top of the pens. Six hours after mixing, there was a significant effect of the PAP block treatment on the occurrence of social negative behaviour (PAP mean; median [95 % CI] for median = 3.6; 2.7 [1.5-5.4] % vs. Control 8.3; 8.7 [6.1-11.3] % *P* = 0.003, social positive behaviour (PAP 5.5; 5.1 [4.5-6.8] % vs. Control 1.4; 1.3 [0-2.8] % *P* = 0.02), feeding (PAP 9.1; 7.4 [5.5-16.5] % vs. Control 1.6; 0.0 [0-6.1] % *P* = 0.02, drinking (PAP 3.1; 2.9 [2.5-3.9] % vs. Control 0.8; 0.5 [0-1.7] % *P* = 0.04) as well as on the sitting position (PAP 1.2; 0.9 [0-2.4] % vs. Control 4.1; 4.2 [2.1-6.0] % *P* = 0.02). Except for social positive behaviour (PAP 5.6; 6.0 [3.9-7.5] % vs. Control 2.9; 2.8 [1.6-4.2] % *P* = 0.02) those differences were not significant 24 h post-mixing anymore. The prevalence of wounded animals was not significantly different between treatments. The PAP in block form may be a promising tool to reduce food neophobia and aggression after mixing. Still, further research is needed to increase the effect of the PAP block over time under commercial farming conditions.

## Findings

There are several types of pheromones. The sex pheromone released by the boar induces immobility in the sows during oestrus and its synthetic analogue has been widely used on commercial farms to identify oestrus females for insemination [1]. The pig appeasing pheromone (PAP) is another type of pheromone which is found in the sebaceous glands of the mammary chains of the lactating sow. The pheromone is then absorbed by the piglets through the vomeronasal organ, present in the nasal cavity, which transduces the pheromonal signal to the amygdala and hypothalamus [2]. Morrow-Tesch and McGlone [3] found that piglets would not nurse when odours were washed from their mother’s skin. Appeasing pheromones appease the neonates and have shown to be effective also in adult animals (e.g. in dogs [4]; in pigs [5]). Pageat [6] developed a synthetic analogue of the PAP based on a mixture of fatty acids similar in composition to sow skin secretions. Several recent works have studied the effect of the PAP in spray form on the behaviour and physiology of sows and growing pigs during mixing and transport [5, 7–9]. McGlone and Anderson [7] reported a higher average daily gain and an improved feed conversion ratio for pigs with PAP applied to the feeder or snout. Guy et al. [8] found a significant reduction in the level of aggression and skin lesions 24 h after mixing in weaned pigs with PAP applied to the pen walls and sides of the feeders. When applying the synthetic pheromone on the pig’s snout immediately prior to the start of vibration, Driessen et al. [9] reported a decreased in the minimum heart rate during the first hour of the transport simulation. However, sprayed PAP may have practical limitations when used under commercial conditions where block diffusers of PAP would be much easier and realistic. Hence, this preliminary study investigates the effect of the PAP in slow releasing blocks against a control on the behaviour and skin lesions of weaners after mixing on a commercial farm.

## Material and methods

### Ethics statement

The present study did not imply any invasive procedure or treatment to the animals that were housed in compliance with the European Directive (2008/120/EC).

### Experimental procedure

This pilot study was conducted on a commercial farm in Catalunya. Two identical rooms of a commercial weaner building were used. Each room (10 m^2^x 4 m^2^) consisted of 6 identical pens where piglets were housed during the five week weaner period. One new batch of piglets was allocated into the two rooms at once. There were two treatments (PAP block and Control) and each room contained one treatment. For this preliminary study, there were 6 replicates per treatment (6 pens per room). In the PAP treatment, the day before weaning and after washing and disinfection, two PAP blocks (1 block/20 m^2^) (Secure Pig® block, Semiokeys, France) were placed hanging on top of the pens at a […] height of approximately one meter above the floor. The active components of the PAP block are: methyl caprate, methyl laurate, methyl miristate, methyl palmitate, methyl linoleate, methyl oleate. The block should spread the pheromone for 6 weeks continuously starting the diffusion when the block is opened. There was no possibility of pheromone diffusion between rooms. To ensure observer blinding, empty blocks were hanged in the control room exactly in the same way than in the PAP room.

### Animal, housing and general management

Weaner pigs were weaned at approximately 28 days of age and allocated to each room in groups of 28 piglets per pen. Litters from 31 mothers were mixed during weaning following the standard farm management. Males and females were housed in different pens and balanced across both rooms. The pens were located in a building for weaner pigs which consisted of several rooms, each with six pens measuring 1.7 × 3.0 m (0.18 m^2^ per pig). Pens had plastic, fully-slatted floors and solid plastic partitions which prevented any physical contact with animals in neighbouring pens. Temperature within the room was controlled to maintain pigs at a temperature set at 26 °C. In each pen, water was provided from one nipple drinker and feed from two feeders (one creep feeder and one weaner hopper with five feeding spaces). Pigs were fed ad libitum.

### Data collection

Behaviour was recorded by direct observation 6 h post-mixing and 24 h post-mixing. Social behaviour and general activity were recorded by scan sampling adapted from the methodology proposed by the Welfare Quality® (WQ®) protocol for growing pigs on farm [10] which is briefly described here. Pigs were scored as either active or inactive. The behaviours recorded from active pigs are shown in Table 1.

**Table 1:** Behaviour ethogram for direct observations (scan sampling) at + 6 h and 24 h post-mixing (adapted from the WQ® protocol for growing pigs and Mc Guy et al., 2009 [8])

Active behaviour	
Social negative behaviour	Aggressive behaviour, including biting or any social behaviour with a response from the disturbed animal
Social positive behaviour	Sniffing, nosing, licking and moving gently away from the animal without an aggressive or flight reaction from this individual
Feeding	Head in one of the two feeders
Drinking	Mouth on water dispenser
Exploring	Sniffing, nosing, licking all features of the pen or paddock and manipulation of enrichment material
Other active behaviours	All other active behaviours (air sniffing, gazing, walking etc.)
Non-Active behaviour	
Resting	Sleeping (lying down with the eyes shut)

Observations took place on two consecutive days (day 0, 6 h post-mixing and day 1, 24 h post-mixing), with two observations blocks per day for each pen (from 10 am to 12 pm and from 2 pm to 4 pm). Within an observation block, each pen was observed 5 times with a 2.5 min interval between two scans. The number of pigs engaged in each social behaviour category (social negative and social positive) and four general active behaviours (feeding, drinking, exploration and “other”) were recorded. Social behaviour, feeding, drinking, exploration and “other” behaviour were expressed in proportion to the total number of active pigs. The percentage of active pigs was expressed in proportion to the total number of observations (active + resting animals). Resting posture was recorded at the same time, by means of the same scan sampling method and counting the number of pigs lying and sitting. Moreover, when pigs were lying a determination was made as to whether they were lying ventrally or laterally. The percentage of pigs huddling was assessed before starting the scan sampling. Huddling was considered when a pig was lying with more than half of its body on top of another pig.

Skin lesions were evaluated individually in each pen on day 1 (+24 h) and day 7 and following the three- point scale described in the WQ® protocol for growing pigs on farm [10]. Pigs were encouraged to stand up in order to make the body more clearly visible. One side of the pigs’ body was inspected visually for the presence of scratches, considering five separate regions: i) ears; ii) front (head to back of shoulder); iii) middle (back of shoulder to hindquarters); iv) hindquarters; and v) legs (from the accessory digit upwards). The tail zone was not considered here. Animals were considered moderately wounded when presenting more than 4 scratches on any body region. Animals were considered severely wounded when presenting more than ten scratches on at least two body regions or any region with more than 15 scratches. Only scratches longer than 2 cm were considered. The percentage of pigs moderately or severely wounded was expressed on the total of pigs housed in each pen.

All observations were carried out by a single observer previously trained to apply the WQ® protocol for growing pigs on farm.

### Statistical analysis

The pen was the statistical unit for all data. Behavioural data were analysed by means of nonparametric generalized estimating equation models using the GENMOD procedure for repeated measures. The models accounted for the effect of the treatment and the day as well as their interaction day x treatment. A Mann–Whitney Wilcoxon test was used to test whether the prevalence of wounded animals in the control and the PAP block treatments were significantly different. A P-value of 0.05 was considered significant for all analyses. The data was analyzed using the statistical package SAS (SAS.9.2.Institute Inc., Cary, NC, USA).

## Results

### Behavioural data

Table 2 shows the mean frequencies of active behaviours and resting postures recorded during the scans for pigs in the PAP block treatment and in the control group. Six hours after mixing, there was a significant effect of the PAP block treatment on the occurrence of social negative behaviour, social positive behaviour, feeding, drinking as well as on the sitting position. Except for social positive behaviour those differences were not significant anymore 24 h post-mixing. No significant differences were observed for exploratory activities, total activity and lying pigs neither 6 h nor 24 h post-mixing. The percentage of huddling animals was similar between treatments, as well as the percentage of animals lying in a ventral position. Significantly fewer pigs adopted a sitting position in the PAP block treatment at 6 h post-mixing compared to the control group.

**Table 2:** Mean occurrence, median and 95 % confidence interval for median (95 % CI) of active behaviours and resting postures recorded during the scan sampling applied in pigs from the PAP block and control pens

6 Hours post-mixing							
Social behaviour and other active behaviours (%)	PAP in block			Control			*P*-value
	Mean	Median	95 % CI	Mean	Median	95 % CI	
Social negative	3.6	2.7	1.5-5.4	8.3	8.7	6.1-11.3	0.003
Social positive	5.5	5.1	4.5-6.8	1.4	1.3	0-2.8	0.02
Feeding	9.1	7.4	5.5-16.5	1.6	0	0-6.1	0.02
Drinking	3.1	2.9	2.5-3.9	0.8	0.5	0-1.7	0.04
Exploration	12.6	12.5	9.1-15.6	11.2	10.1	7.7-15.6	0.8
Other	66.6	65.4	62.0-70.5	76.7	77.3	75.2-82.0	0.043
Active total	77.6	85.1	71.4-90.0	68.4	71.4	53.6-87.5	0.42
Resting posture (%)							
Lying total	27.6	23.4	13.3-33.3	43.9	42.0	16.9-67.8	0.20
Lying ventrally	89.2	87.4	81.2-100	94.5	100	81.2-100	0.11
Sitting	1.2	0.9	0-2.4	4.1	4.2	2.1-6.0	0.02
24 Hours post-mixing							
Social behaviour and other active behaviours (%)							
Social negative	6.6	6.0	2.5-10.5	8.2	7.8	2.9-13.9	0.52
Social positive	5.6	6.0	3.9-7.5	2.9	2.8	1.6-4.2	0.02
Feeding	23.1	20.5	12.9-37.2	17.2	17.3	8.3-28.7	0.25
Drinking	2.4	2.5	1.0-5.0	2.0	1.7	0-3.9	0.50
Exploration	7.9	8.4	6.6-11-6	6.9	6.7	3.5-11.6	0.56
Other	54.4	52.5	44-65.4	62.8	62.4	50-75	0.22
Active total	41.1	43.8	22.1-57.8	42.0	38.8	21.4-58.9	0.91
Resting posture (%)							
Lying total	66.6	67.4	48.2-84.3	68.8	75.0	52.7-87.5	0.88
Lying ventrally	83.0	85.9	75-88.6	81.4	78.5	74.6-91.6	0.51
Sitting	0.17	0.0	0-0.7	0.69	0.0	0-2.7	0.20

### Wounds on the body

Twenty four hours post-mixing, the prevalence of moderately wounded animals (PAP mean; median [95 % CI] for median = 19.5; 17.8 [14.3-25.0] % vs. Control 21.4; 21.3 [10.7-32.1] %, *P*-value = 0.8) and severely wounded animals (PAP 4.8; 3.6 [0-10.7] % vs. Control 9.3; 7.1 [3.6-17.9] %, *P*-value = 0.2) did not differ between treatments. On day 7, there was not a treatment effect either on the prevalence wounded animals.

## Discussion

In livestock species developing an easy and practical way to spread pheromones is necessary for this product to be used as a strategy to improve the welfare of animals under commercial conditions. When applied in one shot application on the feeders of each pen, the PAP in spray form has shown to be efficient in reducing the levels of aggression in pigs [8]. The PAP in block tested in the present study looks for a much easier way to use such pheromone with a longer diffusion at room level than the spray form.

PAP in block resulted in a significant reduction in social negative interactions such as aggression just after mixing (6 h post-mixing). However, this effect was not observed significantly at +24 h post-mixing. Guy et al. [8], reported a significant effect of sprayed pheromones on the level of aggression over 24 h after mixing. McGlone and Anderson [7] reported a similar level of reduction in aggression which sustained over a 48-h period post-mixing. Both former studies applied PAP sprayed on the walls and the feeders. The increase in social positive interactions in the PAP treated pens may indicate that PAP facilitates the formation of stable social interactions more quickly by replacement of social negative interactions by more peaceful social manipulation. This effect was sustained at 24 h post-mixing. Looking at other types of pig-directed behaviours over a 24 h observation period after mixing, Guy et al, [8] did not find a significant effect of the pheromone treatment on tail biting behaviour and belly nosing. However, on day 7 after mixing the frequency of mounting behaviour was higher in pheromone-treated pens. These findings would support the hypothesis that the PAP would be especially useful in facilitating the formation of a stable social hierarchy. Despite a strong effect on social negative interactions, the prevalence of wounded animals was not significantly affected by the treatment in the present study. Still, a slight decrease of severely wounded animals can be noted in the PAP treatment at 6 h post-mixing. Guy et al. [8] and McGlone and Anderson [7] reported a particularly marked effect of pheromone application in the reduction of skin lesions at the front of the body.

Feeding behaviour was strongly affected 6 h post-mixing where the PAP block treatment increased the occurrence of feeding events by almost 9 times. Pigs were also significantly more eager to drink in the PAP block treatment 6 h post-mixing. Those differences were not significant anymore 24 h post-mixing. McGlone and Anderson [7] also recorded that pigs in pens where the pheromone was applied to the feeder spent significantly more time feeding than in the control group.

In terms of resting postures, significantly fewer pigs in the sitting position were observed in the PAP block treatment compared to the control group. Sitting position may indicate a certain lack of comfort and an apathetic state in pigs [11, 12]. Some authors even related sitting position with sickness behaviour (e.g. [13]). In the PAP treatment pens, pigs may feel more “comfortable” and relaxed spending less time in the sitting position. Animals may assume postures that conserve heat when not comfortable or sick, increasing contact with other pigs and adopting a ventral position. The proportion of pigs lying in a ventral position was not affected by the treatment.

As the present pilot study was conducted on a single batch without room replicates, a possible room effect cannot be discarded. Further studies are therefore needed to ensure the robustness of these results.

## Implications

Weaning is a very stressful event on the pig production cycle. The PAP in a block form appeared to help the pigs coping with the new environment increasing feeding behaviour and decreasing the occurrence of social negative interactions, during the six hours post-mixing. The PAP in a block form may be a promising tool to reduce food neophobia and aggression after mixing; still strategies to increase the effect of the PAP in block over time under commercial conditions should be further explored.
